# Value-Based Health Care Delivery, Preventive Medicine and the Medicalization of Public Health

**DOI:** 10.7759/cureus.1063

**Published:** 2017-03-01

**Authors:** Andreas Vilhelmsson

**Affiliations:** 1 Department of Clinical Sciences, Lund University

**Keywords:** value-based health care, public health, personalized medicine, e-health, medicalization, risk, prevention

## Abstract

The real paradigm shift for healthcare is often stated to include a transition from accentuating health care production and instead emphasize patient value by moving to a ‘value-based health care delivery’. In this transition, personalized medicine is sometimes referred to as almost a panacea in solving the current and future health challenges. In theory, the progress of precision medicine sounds uncontroversial and most welcomed with its promise of a better healthcare for all, with real benefits for the individual patient provided a tailored and optimized treatment plan suitable for his or her genetic makeup. And maybe, therefore, the assumptions underpinning personalized medicine have largely escaped questioning. The use of personalized medicine and the use of digital technologies is reshaping our health care system and how we think of health interventions and our individual responsibility. However, encouraging individuals to engage in preventive health activities possibly avoids one form of medicalization (clinical), but on the other hand, it takes up another form (preventive medicine and ‘self-care’) that moves medical and health concerns into every corner of everyday life. This ought to be of little value to the individual patient and public health. We ought to instead demand proof of these value ideas and the lacking research. Before this is in place critical appraisal and cynicism are requisite skills for the future. Otherwise, we are just listening to visionaries when we put our future health into their hands and let personalized solutions reach into people's everyday life regardless of patient safety and integrity.

## Introduction and background

The real paradigm shift for healthcare is often stated to include a transition from delivering 'health care production' to instead emphasize patient value by moving to a ‘value-based health care delivery’ [1-2]. In this context, personalized medicine is sometimes referred to as almost a panacea in solving the current and future health challenges and the impending shortage of healthcare resources; a deus ex machina with medico-technical solutions to master the unequal access to health care and the lack of patient compliance. Personalized medicine is foremost about the key skills involved in the professional relationship with the patient. This means for instance as a physician to take time and be open and listen to the patient and to let the patient explain [3]. At the same time, personalized medicine also builds on the Human Genome Project, which was forecasted to revolutionize disease risk prediction [4]. The individual human genome information now allows providers to create optimized care plans at every stage of a disease, shifting the focus from reactive to preventive health care. By using specific diagnostic tests, doctors can predict how well a patient respond to treatment for some diseases or conditions and advances in genomic and clinical science have created innovative opportunities to further tailor health care to each patient. 

Personalized or precision medicine also maintains that medical care and public health will be radically transformed by prevention and treatment programs more closely targeted to the individual patient. These interventions will be developed by sequencing more genomes, creating bigger biobanks and linking biological information to health data in electronic medical records. One important aspect of personalized medicine is therefore the emerging focus on so-called eHealth solutions to include patients in their personal care; a paradigm shift with the aim to enable patients increased access and influence over their health situation by emphasizing “patient authorization/transparency/empowerment”. Patients who seek a clearer understanding of their disease, its prognosis, and the most effective treatment in terms of efficacy can through personal eHealth options individually monitor their health from home, by medical equipment or with access to medical health records online or through specific smartphone applications for reporting side effects of drugs [5].

## Review

Basically, the promise of precision medicine entails that an individual’s genetic information will be increasingly used to prioritize access to health care in contrast to a “one-size-fits-all” approach, in which disease treatment and prevention strategies are developed for the average person with less consideration for the differences between individuals. By using specific genetic diagnostic tests, doctors may predict how well a patient respond to treatment for some diseases or conditions and enable them to determine the best dosage and duration of treatment according to the genetic setup of the individual patient. In theory, the progress of precision medicine sounds uncontroversial and most welcomed with its promise of a better healthcare for all, with real benefits for the individual patient, provided a tailored and optimized treatment plan suitable for his or her genetic makeup. And maybe, therefore, the assumptions underpinning personalized medicine have largely escaped questioning.

An obvious problem not being discussed is that the genetic tests supposed to revolutionize health care, do not yet exist and to actually run healthcare according to this notion means that a wider group of doctors now will use patient's genetic and other molecular information as part of routine medical care. But many doctors are simply not qualified to make sense of genetic tests or to communicate the results accurately to their patients. Hence, this precision approach may lead to too many false positives and risk of overdiagnosis instead [4]. These aspects are further strongly connected to overmedicalization [6] as the definition of abnormality broadens [7] and where incorrect diagnoses can lead to inappropriate patient care, poor patient outcomes and increased costs [8].

While public health medicine has long engaged in strategies of disease prevention and health promotion, more individualized practices of risk are now argued to have become a central dimension of the politics of life in the twenty-first century [9]. As definitions broaden and thresholds fall, people with smaller risks or milder problems are labeled, which means the potential benefits of treatment decline, raising the possibility that harms will outweigh benefits. Not only may it lead to adverse effects of unnecessary labeling and harms of unwanted tests and therapies for the individual, for society, there is the expense of unnecessary treatment and the diversion of scarce resources away from people who need it, to those who essentially do not. Increasingly we have come to regard simply being at risk of future disease as being a disease in its own right [10]. Diagnostic labels now go beyond the disease itself to include risk factors for disease, sometimes giving rise to a new source of social identity, namely a 'pre-disease' [11]. Electronic health records are often optimistically described as containing a rich database of clinical information and that the future algorithms will be developed to identify patients with disease risk factors or with a need for guideline-based screening, adding to the risk behavior. Less commented is the risk of data breaches of protected health information and overall safety aspects [12] and how people's personal data are being used beyond their private rationales for commercial, governmental, managerial and research purposes [13]. A self-serving medical industry promoting constant monitoring and unnecessary intervention risk leading to a culture that treats everyone as a potential disease, turning healthy people into patients, a kind of medicalization of the "worried well" [14] and on the population basis, a kind of "public healthification".

Focusing on preconditions for disease may further increase what the German sociologist, Ulrich Beck, has called the “risk society” [15] and in a global approach “world risk society” [16]; a society structured through individualization where a social crisis appears as individual crises, no longer perceived in terms of their rootedness in the social realm. But by trying to assess potential risk factors for disease and disorders at earlier stages, the concepts of illness and risk may become increasingly blurred [17]. In that sense, health can be paradoxically both more biomedicalized through processes as surveillance, screening, and routine measurements of health indicators done in the home and seemingly less medicalized as the key site of responsibility shifts from the professional physician to include collaboration with or reliance upon the individual patient/user/consumer [18]. What we have seen is an eruption of "diagnostic" testing offered by independent labs at significant financial and sometimes emotional expense to the patient. All of this "science" has meant that a lot of things can be tested for, but more research is needed to understand what to do with these results to improve patient outcome.

All mentioned aspects are way too important to be disregarded when discussing the transition to value-based health care delivery and personalized medicine. As new eHealth techniques and systems are developed and deployed, new areas of concern and obstacles surface. In a digital risk society [19] aspects such as trust, equality and vulnerability issues, changed power distribution, patient integrity and safety are important issues of concern. Hence, the aspect of personalized health can entail more focus on the individual responsibility of health and with that follow the risk of a more medicalized discourse and not less as indented. This will further drain resources from important public health projects when expensive precision medicine initiatives are in the spotlight and attract research funding as a scientific promise to mankind needing expensive medico-technical solutions. A discussion is therefore needed to assess the actual potential (and not the promises) of precision medicine and what this development means for value-based healthcare and for public health.

## Conclusions

The use of personalized medicine and the use of digital technologies is reshaping our health care system and how we think of health interventions and our individual responsibility. However, encouraging individuals to engage in preventive health activities possibly avoids one form of medicalization (clinical), but on the other hand, it takes up another form (preventive medicine and ‘self-care’) that moves medical and health concerns into every corner of everyday life. This ought to be of little value to the individual patient and public health. We ought to instead demand proof of these value ideas and the lacking research. Before this is in place critical appraisal and cynicism are requisite skills for the future. Otherwise, we are just listening to visionaries when we put our future health into their hands and let personalized solutions reach into people's everyday life regardless of patient safety and integrity. 
